# A multiple drug loaded, functionalized pH-sensitive nanocarrier as therapeutic and epigenetic modulator for osteosarcoma

**DOI:** 10.1038/s41598-020-72552-z

**Published:** 2020-09-23

**Authors:** Ye Yuan, Jia-Xing Song, Mei-Na Zhang, Bao-Shan Yuan

**Affiliations:** The Department of Medicine Laboratory, The First Hospital, Jilin University, No.1 Xinmin Street, Changchun, 130021 Jilin People’s Republic of China

**Keywords:** Bone cancer, Drug delivery

## Abstract

Osteosarcoma is a malignant condition affecting adolescents and children more than adults. Nanobiomedicine has opened up several avenues which have increased therapeutic efficiencies than the conventional treatment for the same. In the current study, a novel organic nanoparticle was devised conjugated with bisphosphonate zoledronic acid which has an affinity for bone tissues. Moreover, the nanoparticle was loaded with multiple anti-cancer drugs like gemcitabine and epirubicin. The nanoparticles were characterized by microscopic analysis, entrapment and loading efficiencies, bone affinity studies, in-vitro release studies, cytotoxicity studies and finally in-vivo tumor regression studies. Bone affinity studies depicted a high affinity of zoledronic acid towards bone powder. The nanoparticle exhibited a nanosize dimension, high entrapment and loading efficiencies with uniform symmetry devoid of agglomeration. The in-vitro release experiments showed a measured release of drugs over a longer time without any hint of burst release. However, the release was comparatively for a longer duration in acidic pH and normal physiological pH which may be excellent for therapeutic efficiency. The cytotoxicity studies revealed enhanced cytotoxic effect for MG-63 cell lines in comparison of free drug or single drug combinations. Nonetheless, they proved to be cytocompatible with primary bone cells. Additionally, cellular uptake of nanoparticle was appreciably improved. Significant tumor (250%) regression was seen upon treatment with multiple drug loaded zoledronic acid conjugated nanoparticle, along with epigenetic changes affecting microRNA expressions. The increased cytotoxicity and increased cellular uptake may be of greater advantage in systemic osteosarcoma therapy. Combining all results, our study demonstrated substantial potential towards management of osteosarcoma.

## Introduction

Nanobiomedicine, in today’s world, offers new techniques in developing novel therapeutics and diagnostic procedures for all known pathologies and complications^1,2^. Additionally, it also helps creating new drug/therapeutic delivery vehicles to targeted tissue for achieving better therapeutics^3–6^. This has genuinely revolutionized the field of medicine for treatments of carcinomas. The advantages of nanoscience are manifold: increased therapeutic efficacy, small quantity requisite, increased uptake of the drug by specific cells, non-clearance of drug from the circulation owing to small size, better balance between the side effects & treatment efficacy and a targeted delivery.

Bone defects and tumours include a huge range of several skeletal disorders which impairs mobility in humans in addition to causing death^1^. Significant amongst these is osteosarcoma, a very common malignant bone tumor which mostly affects the adolescents and children^7^. Although the last decade has witnessed great changes in the management and treatment of osteosarcoma, the recovery rate is still 65–70%^8^. Unfortunately for the rest 30–35%, the treatment is associated with toxic side effects^9^. Biphosphonates are a cluster of drugs which are reported to lessen bone erosion and the hazards of bone osteoporotic impediments safely and effectively^10,11^. They have also been known to reinstate bone mass and homeostasis^12^. Encompassing all these unique features, bisphosphonates were attempted to conjugate with nanoparticles in several studies^10,13^ to gain osteotropicity for treatment of osteoporosis. Amongst them, zoledronic acid is a bisphosphonate that contains nitrogen which was demonstrated to have the potential to act as an anticancer agent^14^.

Therefore, in our current study, we conjugated zoledronic acid with PLGA nanoparticles which were loaded with multiple anticancer drugs namely, gemcitabine and epirubicin. PLGA was the choice of organic nanocarrier owing to its good biocompatibility and degradability^15,16^. Gemcitabine (2′, 2′-difluorodeoxycytidine) is used as first line treatment for cancer frequently^17^. Epirubicin, on the other hand, is an anthracycline anti-cancer drug which was chosen because of its lower toxicity profile^18,19^. It has been viewed that nanotherapeutics for cancer with single drug was not able to produce satisfactory results^20^. Therefore loading multiple chemotherapeutic drugs with similar properties could act synergistically^21^ to lead to development of a novel therapy for osteosarcoma. The study aimed at investigating the synthesis and characterization of a novel nanocarrier along with its cytotoxicity caused by multiple drug regimen loaded in PLGA nanoparticle as well as effects of conjugating zoledronic acid with the PLGA nanoparticles. Additionally, it also aimed to evaluate the novel nanocarrier for its pH sensitivity through the in-vitro release kinetics studies too.

## Materials and methods

### Materials

PLGA 502H, ratio of lactide/glycolide being 50:50 while the inherent viscosity was 0.22 dl/g was obtained from Boheringer Ingelheim, Germany. Poloxamer 188, HCl, NaOH, SDS, DMF and DMSO were procured from Sigma Aldrich, China. DMEM and RPMI 1640 media, FBS, PBS, penicillin, streptomycin, trypsin, EDTA, HBSS were acquired from GIBCO, China. 3-(4,5-Dimethylthiazol-2-yl)-2,5-diphenyltetrazolium bromide (MTT) and Propidium Iodide (PI) were acquired from Sigma Aldrich, China. The anti-cancer drugs gemcitabine and epirubicin along with zoledronic acid were from Sun Pharma advanced research centre, India. All the tissue culture and cell plates were procured from NUNC, Finland. Human bone particles were obtained from Merck research laboratories for research use and no approval/clearance was needed for use of this research material, as suggested by the Jilin University Committee on use of Human subjects/materials. No information identifying the subjects was provided by the vendor and no consent was needed for our research study as this was commercially available product without such requirements/limitations.

### Synthesis of PLGA-zoledronic acid

The procedure of ZOL conjugation with PLGA was executed utilizing a conjugation linker, N, N′-Carbonyldiimidazole (CDI) in this case. The procedure was adapted from Chaudhuri et al.^10^ with definite modifications. Briefly, zoledronic acid (ZOL, 100 mg) and distilled DMF were mixed together with triethylamine (TEA). 90 g of moisture free CDI was further mixed with the resultant solution in firmly shut vessel with nitrogen atmosphere at 60 °C for 24 h continuously. Acetonitrile was utilized to wash the precipitates twice after TEA was evaporated. Following this procedure, PLGA and activated ZOL were dissolved in the ratio of 1:22 (w/w) in DMSO firmly secured vessel in nitrogen atmosphere with TEA and reaction was allowed for 12 h. Dialysis of the reaction mixture was carried out with distilled water for removal of surplus activated ZOL.

### Synthesis of multiple drug loaded PLGA-ZOL nanoparticles (NPs)

Solvent diffusion or nano-precipitation was the preferred method for the synthesis of multiple drug loaded PLGA-ZOL NPs. The procedure followed was adapted from Fessi et al.^22^ with minor changes. Acetone was the chosen organic phase with gemcitabine and epirubicin (7.5 mg each) and PLGA-ZOL (100 mg) were gradually added into 20 ml of aqueous phase at the rate of 0.5 ml/min. The aqueous phase comprised of Poloxamer 188 (0.5% w/v) as stabilizer with a magnetic stirrer. The recovery of the NPs was carried out by centrifugation at 25,000 rpm for half an hour followed by lyophilization with trehalose as a cryoprotectant for 48 h. The NPs hence prepared with dual drug was termed PZ-3. The PLGA-ZOL NPs were also prepared loaded with single drug, ones prepared with only gemcitabine was PZ-1, ones with only epirubicin was PZ-2. Blank PLGA-ZOL NPs without any drugs were prepared too.

### In-vitro bone binding studies

Zoledronic acid solution and PLGA-ZOL NPs were studied for their in-vitro bone affinity. Human bone particles (125 mg) with sizes from 160 to 185 μm were washed two times with binding buffer, 0.2 M Tris–formate at pH 7.4. Equal amount of concentration of both zoledronic acid and PLGA-ZOL NPs were diluted with PBS and were continuous stirred with human bone powder for 6 h in binding buffer, the final volume of which was adjusted to 100 μl at 22 °C. Every reaction was terminated by application of vacuum. The resultant solution was washed three times with binding buffer and once with ethanol, each of 100 μl. Bound bone particles were then exposed to scintillant. At regular time interval of 1, 3 and 6 h, binding with human bone powder was calculated by Microbeta JET by Perkin Elmer. The procedure was adapted from an earlier report^23^.

### Morphological analysis of NPs

The morphology of PLGA-ZOL NPs PZ-1, PZ-2, PZ-3 was observed through transmission electron microscope (JEOL JEM-200CX). Concisely, samples of NPs were retained in a copper grid coated with carbon and counter-stained with phosphotungistic acid along with air drying the whole thing for 2.5 h. The NPs were studied in predictable TEM mode under acceleration voltage of 100 kV.

### Size distribution and stability of NPs

The average size distribution of PLGA-ZOL-NPs, PZ-1, PZ-2, PZ-3 was determined using Malvern zeta sizer (Malvern Instrument Inc). The dilution of the samples of NPs was carried out with PBS followed by uniform dispersion with ultra-purified water and the analysis was performed such that the mean count rate stayed near 200. Zeta potential measurements of PLGA-ZOL-NPs, PZ-1, PZ-2, PZ-3 were done. The procedure involved utilizing an aqueous dip cell in an automatic manner where the diluted NPs were inserted in the capillary measurement cell and locus of cell was attuned. The synthesized NPs were deliberated for distribution of particle size and electrical properties as viewed by zeta potential measurements and polydispersity index. The studies were done thrice to maintain reproducibility and the results were deliberated as mean ± SD. The activities of NPs under changed external pH were also studied where they were diluted in PBS (10 mM phosphate and 150 mM NaCl) where the pH ranged from 3.5 to 8.0. The studies were carried out thrice to maintain reproducibility and the data attained was analyzed using the Zetasizer 6.01 software that was delivered with the nano zeta sizer (Malvern Instrument, Inc).

### Encapsulation and loading efficiency

The encapsulation efficiency (EE%) and loading efficiency (LE%) of the drug into the NPs were determined using HPLC method. Filtration of PZ-1, PZ-2 and PZ-3 was done with Amicon centrifugal tube whose molecular cut-off was 3000. The amount of free drug or non-entrapped drug from the remnants of the total drug added was determined from the filtrate. The filtrate (10–12 µl) was inserted into the HPLC column. Previously, separate calibration curves for epirubicin and gemcitabine were determined. Calculations were made by the following equations to define the quantity of drugs in the filtrate

$$  LE\,(\% ) = Weight\;of\;drug\;loaded \div Weight\;of\;the\;drug\;loaded\;NPs \times 100 $$

$$  EE\,(\% ) = Weight\;of\;drug\;loaded \div Total\;amount\;of\;drug\;added \times 100  $$

### In-vitro release of drugs

The procedure was followed from Wang et al.^24^ with necessary modifications for suiting our studies. Dialysis method was the chosen one to evaluate the drug release from PZ-1, PZ-2, PZ-3. Concisely, PZ-1, PZ-2, PZ-3 NPs were freeze dried. The release study was performed in both at pH 7.4 (physiological media; phosphate buffered saline) and pH 4.5 (tumor microenvironment) at room temperature. 1 ml of ultra-purified water was used to disperse the freeze dried NPs. Then they were sealed in the tubing of dialysis membrane, the molecular cut off of which was 3000. The sealed dialysis membrane was retained in 10 ml of release media. This entire assemblage then was submerged in shaking water bath retained at room temperature. At particular intervals of time, 1 ml of the media was removed and renewed with identical volume of new medium. Using HPLC technique, released drug in the media was quantified. The instruments used were Agilent 1100 alongside G1311A pump, a G1314A programmable diode array detector (DAD) and a G1313A auto-injector. Analytical column C18 (250 mm × 4.6 mm ID, 5 μm) was employed for the study. For characterization of the concentration of individual drug released, different mobile phase was engaged.

### Cytotoxicity studies

#### Cell culture

For 2D culture, the MG-63 cell line was obtained from ATCC (China). Cell culture was dne in DMEM (Gibco™, Beijing, China) comprising of 10% fetal bovine serum (FBS) alongside 1% penicillin and streptomycin. MG-63 OS cells were seeded in 12-welled culture plate at 40,000 cells/well 2 days prior to incubation with free drugs and NPs. The incubation of cells was done at room temperature in a saturated environment with 5% CO_2_.

Primary bone cells were obtained from *Wistar* rats which were used for some experiments in other laboratory. However along with the in-vivo tumor study, permission for the work was obtained from University Committee on Animal Ethics. The procedure was followed from Baker et al.^25^. The long bones of the rats were obtained and harvested after which the epiphyses were removed followed by flushing out bone marrow with a syringe and needle. Small fragments of the diaphysis were made which were washed with PBS followed by incubation in 2 mg/ml collagenase II in DMEM at room temperature in a pulsating waterbath for removal of adhering cells. The fragments so obtained were washed with 10% fetal calf serum and cultured in DMEM augmented with 100 U/ml penicillin, 50 mg/ml streptomycin sulphate and gentamycin, 1.25 mg/ml fungizone along with 100 mg/ml of ascorbate. On attaining confluency, 0.25% trypsin and 0.1% EDTA in PBS was used for cell harvesting. They were then plated at 25 × 10^3^ cells per well in 6-well cell culture plates and repeatedly cultured in 3 ml medium mentioned above until confluency.

#### MTT assay

3-(4,5-dimethythiazol-2-yl)-2,5-diphenyl tetrazolium bromide (MTT) assay was employed for measurement of cytotoxicity of the free drugs and NPs. The principle of the assay is utilizing mitochondrial succinate dehydrogenase for reduction of yellow MTT. Insoluble formazan complex was formed after the entry of MTT in the live cells after which it was being reduced. For this, MG-63 and primary bone cells were seeded at a density of 1 × 10^4^ in a 96-well plate. The cells after attaining confluency were treated with PLGA-ZOL NPs, free epirubicin, free gemcitabine, PZ-1, PZ-2, PZ-3 NPs. The drug concentration of the PZ-1, PZ-2, PZ-3 were adjusted to the free drug concentration which was 2.5 µg/ml. The cells were gestated for 24 and 48 h consequently. After a certain time, the cell culture plates were treated with 100 μl of MTT solution (5 mg/ml) and incubation was done for 4 h as per Wang et al.^24^. Addition of DMSO led to the extraction of the formed formazan crystals and were incubated for 30 min further. Absorbance at 570 nm was read using a microplate reader (ThermoFischer, China) for each plate. The experiments were repeated for 6 times to maintain reproducibility.

#### Alamar blue assay

Cytotoxicity of the PLGA-ZOL NPs, free epirubicin, free gemcitabine, PZ-1, PZ-2, PZ-3 NPs were also assessed by Alamar blue assay^13^. MG-63 cell culture was done with DMEM medium at density of 10^4^ cells in each well of 96-well plates. This was augmented with 10% FBS in a 5% CO_2_ humidified atmosphere at 37 °C within an incubator for 24 h. Replacement and renewal with fresh medium was carried out. The medium was incubated with following samples: PLGA-ZOL NPs, free epirubicin, free gemcitabine, PZ-1, PZ-2, PZ-3 NPs. The drug concentration of the PZ-1, PZ-2, PZ-3 were adjusted to the free drug concentration which was 2.5 µg/ml. After 72 h, removal of medium and rinsing with PBS was done followed by the addition of 250 µl of Alamar blue solution (the ratio being 10:10:80 Alamar blue: FBS: medium 199 v/v) and further incubation was carried out for 3 h. 300 µl of Alamar blue solution was removed into a new 96-well plate and reading was noted with automated microplate spectrophotometer (EPOCH Microplate Spectrophotometer, USA) at 530 nm and 590 nm (excitation and emission wavelength respectively. the same process was repeated with primary bone cell culture.

#### IC_50_ detection

The concentration of sample ensuing 50% cell death is IC_50_ of the sample which was deliberated from concentration-effect curves, in view of the optical density of the control well (cells treated with PBS) as 100%.

IC_50_ is determined using the following formula:

$$  {\text{Inhibition of}}\;50\% \;{\text{growth of cells}}\,({\text{IC}}50) = {\text{OD control}} - {\text{OD treated cell}} \times 100 \div {\text{OD control}} $$

The measurements were repeated thrice.

### Cellular uptake of the NPs

The technique was modified from an earlier report^26^ with appropriate changes to suit our study. 5 × 10^4^ cells/ml concentrations of MG-63 were harvested in 100 mm tissue culture plates. Incubation of cells were done at room temperature for 48 h with free epirubicin, free gemcitabine, PZ-1, PZ-2, PZ-3 NPs at the earlier calculated IC_50_ concentration. Following this, washing of the cells was done with PBS twice followed by trypsinization and resuspension in PBS. The amounts of drug amassed in the cells were determined with an inductively coupled plasma mass spectrometer (ICP-MS, ELAN DRC-II, PerkinElmer). In brief, 23% nitric acid treatment of the cell suspensions were carried out followed by 8% H_2_O_2_ and wet autoclaving of cell suspension was done for 1 h. This was then inducted in the ICP-MS for quantification of amount of drug uptaken by the cells.

### In-vivo anti-tumor studies

#### Animals

Male *Sprague–Dawley* rats weighing upto 320–380 g were utilized in the current study. All experiments were undertaken in 7–10 week old male rats. The Jilin University Committee on Animal Ethics granted permission for the animal experiments via its approval letter (6134, dated Jan 14, 2019). All experiments were performed as approved by the committee and all methods were carried out in accordance with relevant guidelines and regulations. 3 rats were used for a single group for all experimental work.

#### Anti-tumor studies

Determination of better therapeutic efficacy of the PZ-1, PZ-2, PZ-3 NPs compared to free drugs was done by the in-vivo studies. Tumors were grown in *Sprague–Dawley* rats by injecting MG-63 OS cells (1 × 10^5^) in them. The conventions were appropriately modified from Eloy et al.^27^, Kempf et al.^28^ and Tomoda et al.^29^. Tumors would grow to a noticeable size after 10–14 days of beginning of the treatment. Dilution of PZ-1, PZ-2, PZ-3 NPs, PBS being negative control and epirubicin long with gemcitabine being positive controls were done in PBS buffer at pH 7.4 before administration. The dose of epirubicin and gemcitabine administered are 2.0 mg/kg/day twice a week for 4 weeks. The drug concentration of the PZ-1, PZ-2, PZ-3 were adjusted to the free drug concentration which was 2.0 mg/kg/day. The groups made were as follows: (I) PBS (negative control); (II) Free epirubicin; (III) free gemcitabine; (IV) PZ-1; (V) PZ-2; (VI) PZ-3 (n = 5 in each group). Until the end of the study, tumor volume and body weight were noted for all tumor-bearing mice following which they were euthanized by anaesthetics overdose. The length and width of the tumors were measured with a calliper and documented every 2 days. Calculation of tumor volume was done according to the equation: (width^2^ × length)/2. For ease of comparison within groups, at each measurement point, relative tumor volume was deliberated.

#### Quantitative RT-PCR

RNAs were extracted from tumor using Qiagen kits (Qiagen, Shanghai, China) and suspended in nuclease-free water. Quantitative PCR was performed using an ABI7300 machine (Applied Biosystems, China). miRNA levels were determined, relative to U6, using the SYBR Green PCR kit (Qiagen, China). Fold change in expression was determined by the method of cycle threshold (CT) using the formula of 2^−ΔΔCT^.

### Statistical analysis

Results are expressed as mean ± standard deviation. We used Student's *t*-test to compare groups.

## Results

### Physical characterization of the NPs

**Figure 1:**
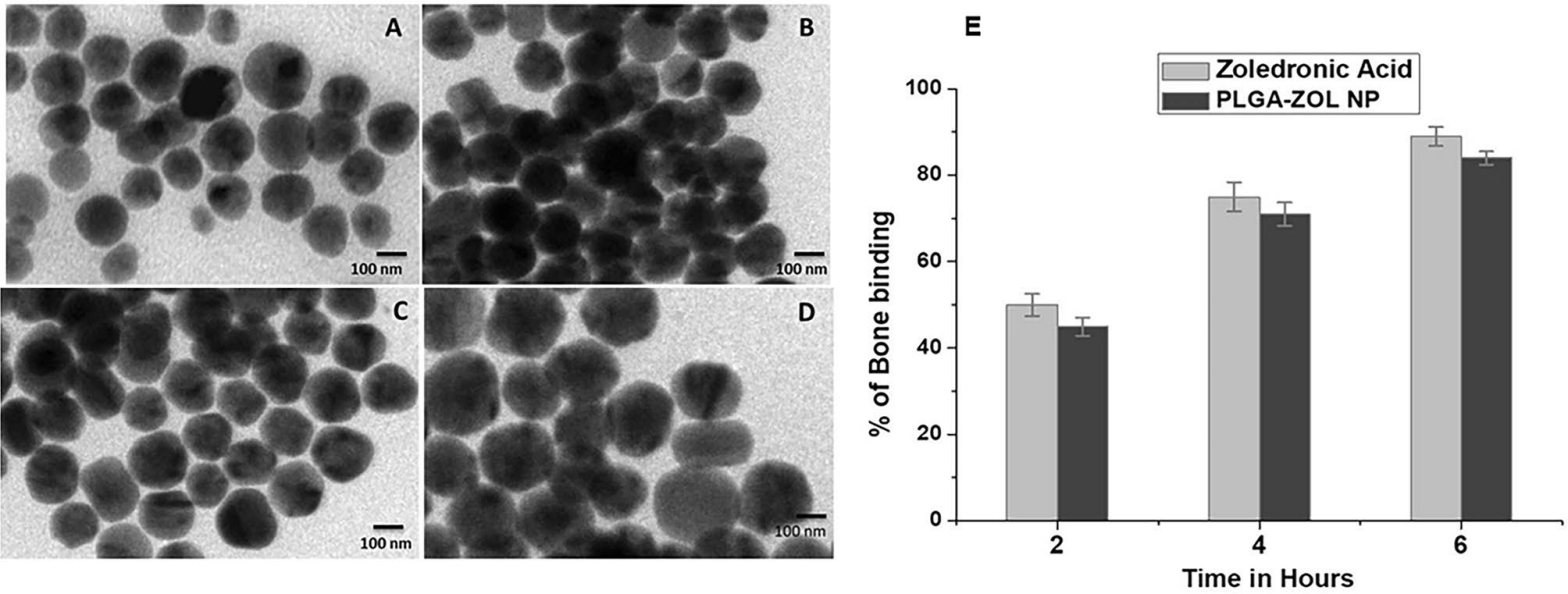
**A**–**D** TEM photomicrographs of NPs; (**A**) PLGA-ZOL NPs; (**B**) PZ-1; (**C**) PZ-2; (**D**) PZ-3. (**E**) Percentage of bone binding exhibited by PLGA-ZOL NPs and zoledronic acid.

Electron micrographic images, as seen in Fig. 1A–D depicted that PLGA-ZOL NPs, PZ-1, PZ-2, PZ-3 NPs have maintained intact shape. They were spherical in shape, not impeccably circular and a little edgier around the corners which may be due to the co-encapsulation of the two anti-cancer drugs. There were no agglomeration of the particles and they seemed uniformly distributed. The sizes of the NPs varied from 142 to 245 nm duly (elaborated in Table 1 along with the zeta potential and PDI). The zeta potential was positive ranging from 22.4 to 26.5 mV. The approximate PDI was in the range from 0.23 ± 0.03, indicating a stable NP formulation.

**Table 1:** Size distribution, zeta potential and polydispersity index (PDI) of the NPs.

Sl. no.		Size of nanoparticles (nm)	Zeta potential (mV)	PDI
1	PLGA-ZOL NPs	142.92 ± 1.2	17.6 ± 0.1	0.211 ± 0.03
2	PZ-1	191.13 ± 1.2	22.4 ± 0.3	0.231 ± 0.04
3	PZ-2	195.13 ± 1.6	23.3 ± 0.2	0.262 ± 0.03
4	PZ-3	245.45 ± 1.5	26.5 ± 0.3	0.323 ± 0.01

Under a varied range of pH, the maintenance of structural integrity of multiple drugs loaded PZ-3 NPs was evaluated by calculating their particle size distribution as a function of external pH. PZ-3 NPs demonstrated unimodal regularity in size distribution all through the pH range (3.5–8.0). The diameter of NPs was calculated to be 197 ± 7 nm (PDI = 0.408 ± 0.042) at pH 7.0 and not much variation was seen in other pH solutions. The information on the diameter of the NPs is provided in Table 2.

**Table 2:** Hydrodynamic diameter and polydispersity index (PDI) values of PZ-3.

pH	Hydrodynamic diameter	PDI
3.5	245 ± 5	0.522 ± 0.041
4.0	257 ± 6	0.517 ± 0.024
5.0	220 ± 3	0.463 ± 0.021
6.0	212 ± 5	0.422 ± 0.036
7.0	197 ± 7	0.408 ± 0.042
8.0	150 ± 6	0.373 ± 0.034

### In-vitro bone affinity studies

The binding affinity of zoledronic acid solution and PLGA-ZOL NPs was high. The results depicted in Fig. 1E shows high affinity of both of them towards bone powder. Zoledronic acid after 6 h was 89% absorbed in bone powder whereas 84% of PLGA-ZOL NPs was found localized in bone powder.

### Encapsulation and loading efficiency

The EE of PZ-1 was 80.2 ± 5.3% whereas the LE was 27.3 ± 3.2%. For PZ-2 the EE was 82 ± 2.3% and LE was 29.2 ± 2.4%. For PZ-3, the EE and LE were 89 ± 4.5% and 33.2 ± 4.4% respectively. The results presented in Table 3 clearly demonstrated high LE and EE for all the NP formulations.

**Table 3:** EE (%) and LE (%) for all the NP formulations.

Sl. no.	NP formulations	EE (%)	LE (%)
2	PZ-1	80.2 ± 5.3	27.3 ± 3.2
3	PZ-2	82.4 ± 2.3	29.2 ± 2.4
4	PZ-3	89.8 ± 4.5	33.2 ± 4.4

### In-vitro release of drugs

**Figure 2:**
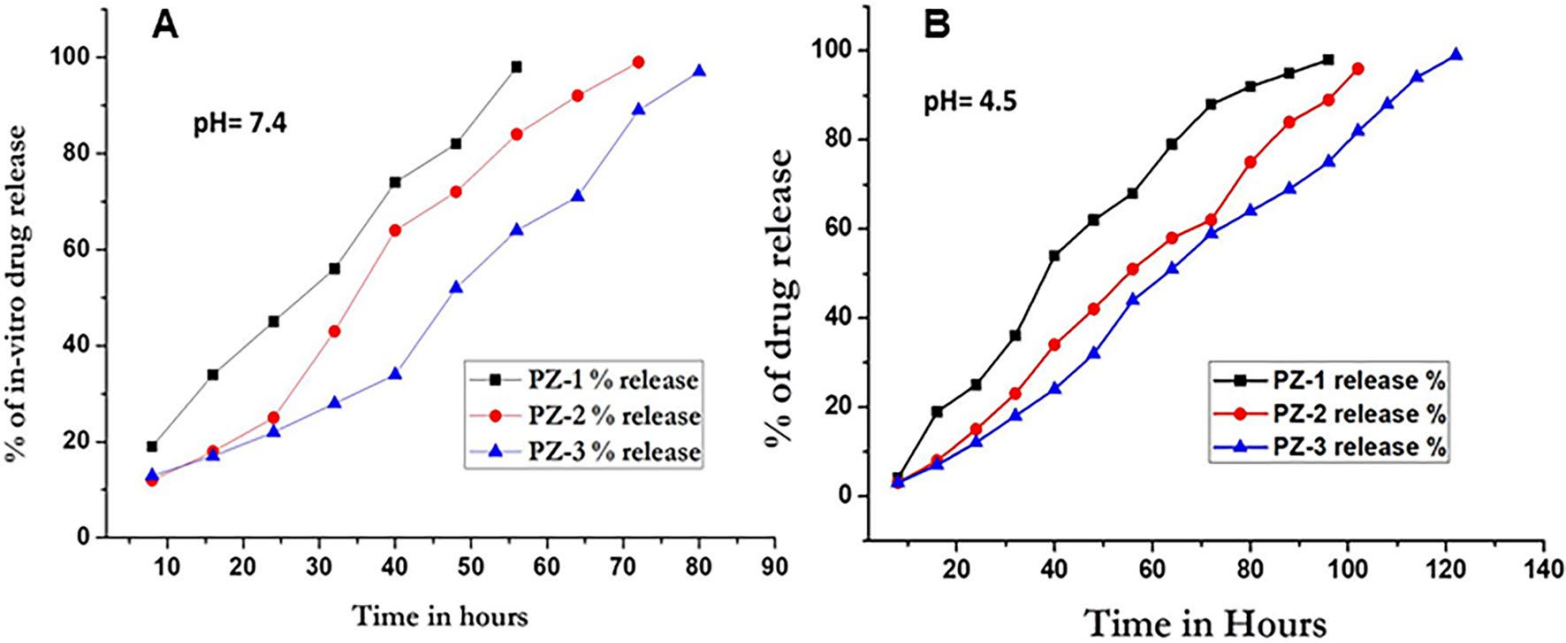
Percentage of in-vitro drug release; (**A**) pH 7.4 and (**B**) pH 4.5.

In PZ-3, gemcitabine and epirubicin were encapsulated with the PLGA-ZOL NP in the ratio of 1:1. However, it was seen that the release profile of gemcitabine and epirubicin in individual NPs or in PZ-1 and PZ-2 are significantly different. In pH 7.4 or physiological milieu, epirubicin diffused faster than gemcitabine at the end of 24 h. It was seen that about 45% of epirubicin (PZ-2) had diffused in comparison of 25% of gemcitabine (PZ-1). The release of drugs from PZ-3 was steady, which lasted upto 80 h. However, in acidic pH (pH 4.5) or tumor microenvironment milieu, the diffusion was slower and sustained. After 24 h, the release of epirubicin (PZ-2) was 25% and gemcitabine (PZ-1) was 15% respectively. The drugs released from PZ-3 continued upto 120 h. This is clearly depicted in Fig. 2.

### Cytotoxicity studies

**Figure 3:**
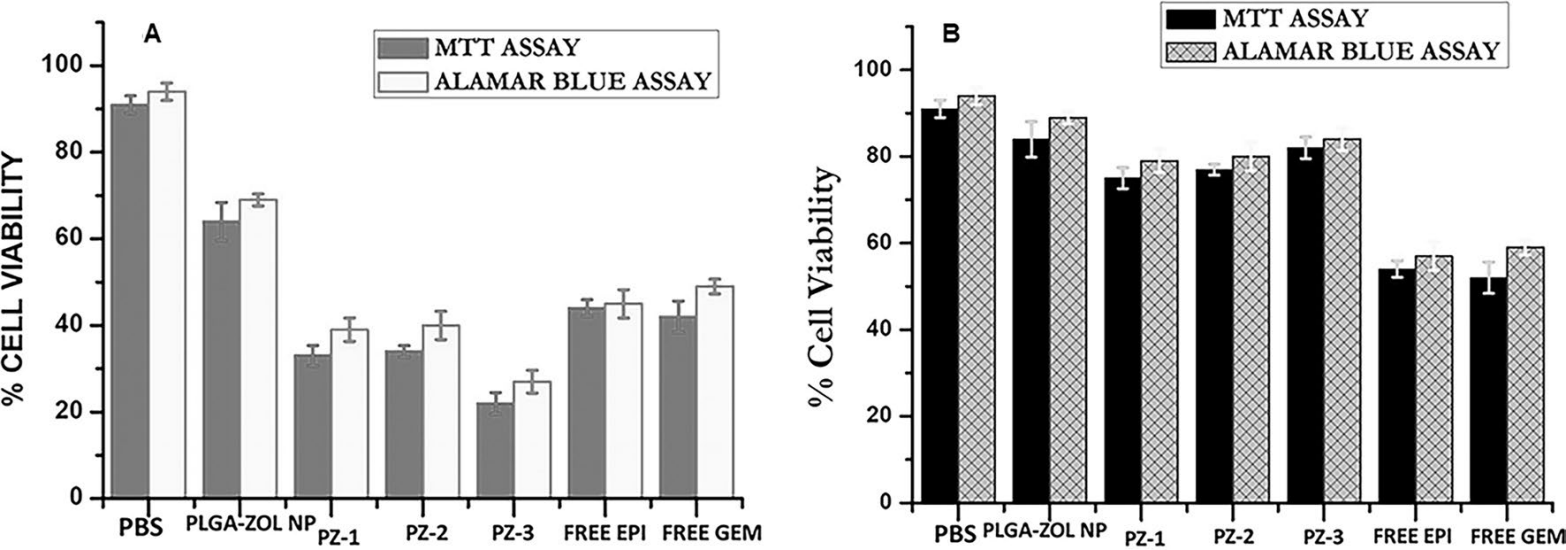
(**A**) Cytotoxicity of MG-63 cells on treatment with PBS, PLGA-ZOL NPs, free epirubicin, free gemcitabine, PZ-1, PZ-2, PZ-3 as exhibited by MTT and Alamar Blue assay. (**B**) Cytotoxicity of primary bone cells on treatment with PBS, PLGA-ZOL NPs, free epirubicin, free gemcitabine, PZ-1, PZ-2, PZ-3 as exhibited by MTT and Alamar Blue assay.

There was noteworthy alteration in cell toxicity between the MG-63 cells treated with PLGA-ZOL NPs, free epirubicin, free gemcitabine, PZ-1, PZ-2, PZ-3 NPs. There was an increase in cell death when the two drugs were co-encapsulated in PZ-3 than alone as indicated in Fig. 3A (MTT and Alamar blue assay). It was seen that after 24 h the decrease in cell proliferation was more in PZ-1, PZ-2, PZ-3 NPs the free drugs. The cell viability percentage in control was 91% and 94% (MTT and Alamar blue respectively) whereas treatment with PLZA-ZOL NPs resulted in 64% and 69% viable cells after 72 h of incubation. The cell viabilities were 33, 34 and 22% for PZ-1, PZ-2, PZ-3 respectively in MTT assay whereas it was 39, 40 and 27% for PZ-1, PZ-2, PZ-3 respectively in Alamar blue assay. The cell viabilities were comparatively much lower than free epirubicin and gemcitabine with 44% and 42% respectively for MTT assay & 45% and 49% for Alamar blue assay. It was calculated that compared to free drugs, the cell viabilities were lessened by 1.33 times in both assays by PZ-1, PZ-2, PZ-3 NPs (Fig. 3A).

With the primary bone cells, the cytotoxicity studies depicted entirely different results, as shown in Fig. 3B. It was seen that PBS as control had the highest number of viable cells (94%) in MTT assay. However, the other formulations (PLGA-ZOL NPs, PZ-1, PZ-2 and PZ-3) had comparable cytocompatibility of 84%, 75%, 77% and 82% respectively. The free epirubicin and gemcitabine had low cytocompatibility of 54% and 52% respectively. In Alamar blue assay of primary bone cells, a similar kind of cytocompatibility was seen (Fig. 3B). It was seen that in MG-63 and primary bone cells, the MTT assay when compared to Alamar blue, was the more sensitive one.

**Figure 4:**
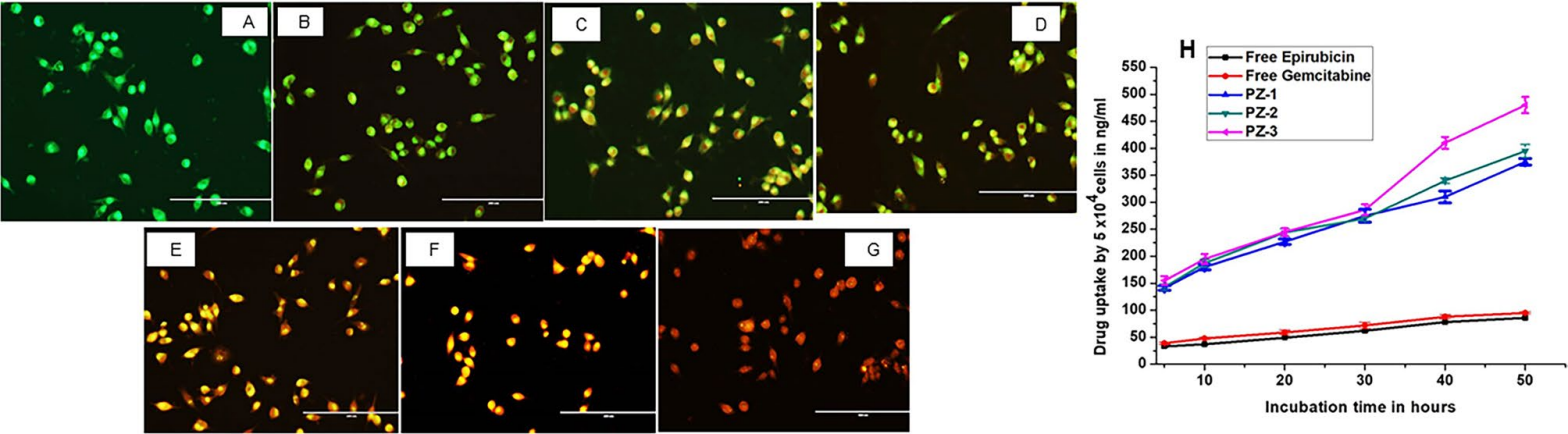
**(A–G)** Cytotoxicity of MG-63 cells as seen by the live dead cell assay. (**H**) Cellular uptake of free epirubicin, free gemcitabine, PZ-1, PZ-2, PZ-3 by MG-63 cells.

The live dead cell assay shown in Fig. 4A–G reinforced the MTT assay results. An important point to be noted is that the cells treated with PZ-1, PZ-2 and PZ-3 (Fig. 4E, F and G) exhibited damaged and broken cells along with dead cells. It was seen in Fig. 4B that the blank PLGA-ZOL NPs did not inhibit cell growth but also did not increase their proliferation. The IC_50_ concentration of the NPs in MG-63 cells is presented in Table 4.

**Table 4:** IC_50_ concentration of the NPs in MG-63 cells.

	IC_50_ value ± S.D
Epirubicin	0.58 µg/ml ± 0.02
Gemcitabine	0.79 µg/ml ± 0.01
PZ-1	1.9 µg/ml ± 0.01
PZ-2	1.67 µg/ml ± 0.02
PZ-3	2.45 µg/ml ± 0.03

### Cellular uptake studies

Figure 4H shows the cellular uptake efficiency of free epirubicin, gemcitabine, PZ-1, PZ-2, PZ-3 NPs. 2.5 µg/ml was the dosage of the free drug concentration which was attuned with the concentration of the drug encapsulated in the NPs. It was seen that cellular uptake of free drugs was significantly less than that of NPs by MG-63 osteosarcoma cell line. The cellular uptake of the PZ-1, PZ-2, PZ-3 NPs were calculated in ng/mol. Accelerated cellular uptake of PZ-1, PZ-2, PZ-3 NPs was evidenced by the MG-63 uptake when compared to free epirubicin and gemcitabine. It was calculated that there was an approximate 6 times increase in the cellular uptake of the NPs as compared to free drugs.

### In-vivo studies

**Figure 5:**
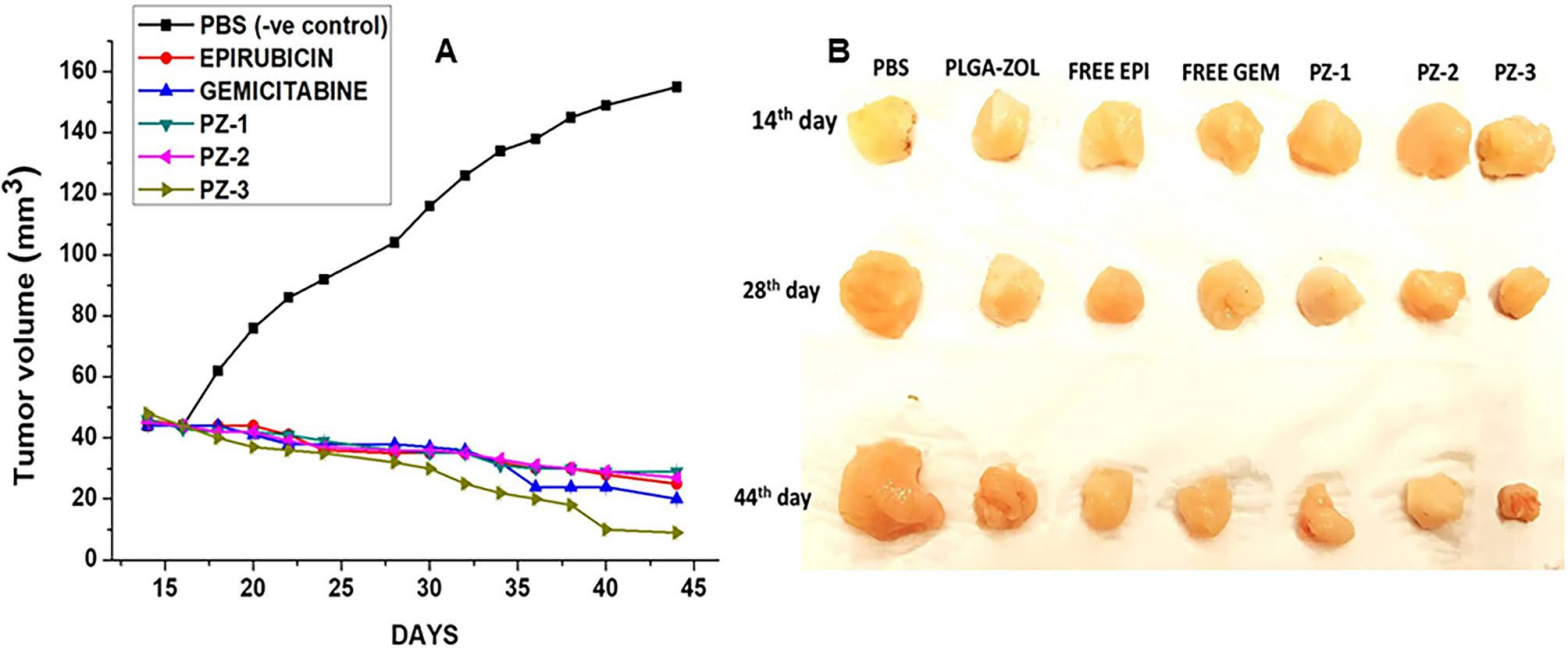
(**A**) Tumor volume obtained from rats treated with PBS, PLGA-ZOL NPs, free epirubicin, free gemcitabine, PZ-1, PZ-2, PZ-3. (**B**) Size of the tumors dissected from rat models treated with PBS, PLGA-ZOL NPs, free epirubicin, free gemcitabine, PZ-1, PZ-2, PZ-3. Note the decreased and restricted tumor size in rats treated with PZ-3.

The anti-tumor studies, as seen in Fig. 5, revealed that the epirubicin solution had less effect in decreasing the size of the tumors than gemcitabine. But the free drugs were still effective in reducing tumor sizes, compared to PBS (control). On the contrary, the tumors were extremely sensitive towards PZ-3 displaying statistically noteworthy reduction in tumor size when treated at 2.5 µg/ml twice a week for 4 weeks. The significance of the PZ-3 where loading of multiple drugs was a tactic to increase the in-vivo anticancer effect was actually in view of the fact that there was almost 250% increased tumor volume on treating with PBS when compared to PZ-3 and the tumor volume decreased by 130% with PZ-1, 115% with PZ-2. This is clearly seen in Fig. 5A, B. It may be important to know that in-vivo multiple drugs loading in PLGA-ZOL NPs enhanced the efficacy of drugs as exhibited by reduction in tumor volume and cellular uptake studies. Lastly, we checked for the miRNA expressions as evidence for epigenetic regulations by PZ-3 and found that oncomiRs miR-21 and miR-10b were downregulated by PZ-3 in the tumors while the tumor suppressor miR-34a was upregulated (Supplementary Fig. 1). Thus, nanoparticles affect epigenetic regulation, which could serve as the possible mechanism of action.

## Discussion

Owing to the perfusion and isolation of bone tissue, the chemotherapeutic advances were never able to attain the concentration required for tumor obliteration. This has paved the pathways for designing of several nano-drug carriers for therapy of osteosarcoma. To name a few important ones, Wang et al. formulated drug loaded magnetic liposomes^24^, Ta et al. made orthophosphate hydrogel from chitosan–dipotassium for doxorubicin delivery for osteosarcoma treatment^30^ and Susa et al. prepared dextran-centred lipid-modified polymeric nanoparticle conjugate for the same^9^. Doxorubicin decorated magnetic liposomes were also tested for osteosarcoma therapeutics^13,31^. However, all of these advances were either associated with cytotoxic effects or were extremely expensive. Therefore, an effective nanoparticle therapeutic moiety with minimal side effects is an unmet and extremely imperative prerequisite of the hour. In our study, zoledronic acid conjugated PLGA nanoparticle would have to be loaded with multiple anticancer drugs and targeted to the specific tissue. There have been instances where it has been demonstrated that combination regimen of drugs that worked well in-vitro does not provide adequate results in-vivo because of the complexity of cancer microenvironment. Therefore, in-vivo studies in rats were carried out too. Interestingly, our data supports epigenetic regulation by the nanoparticles, as determined by miRNA levels in the tumors after treatment with PZ-3, compared to controls. The oncomirs, miR-10b^32^, miR-21^33^ were downregulated by PZ-3 and the tumor suppressor miR-34a^34^ was upregulated. This is in agreement with epigenetic regulations in osteosarcoma^35–37^ that need further evaluations.

It had been already reported earlier that single chemotherapeutic drugs loaded in a nanocarrier may yield unsatisfactory results for osteosarcoma therapeutics due to several factors^20^. Therefore, in our study, a combination of drugs epirubicin and gemcitabine was loaded into an organic nanoparticle synthesised from PLGA. This was done so that after successful drug delivery to the targeted tissue, the nanoparticle may be biodegraded without any toxic side effects. PLGA NPs were of ideal sizes that avoided being cleared by reticuloendothelial system^38^. It was also observed that despite the combinatorial drug theory, there wasn’t a significant improvement in the osteosarcoma therapeutics due to early clearance of drugs from circulation, poor tissue targeting resulting in non-significant accumulation of drugs in the affected bone cells^39^. Therefore we hypothesized that a probable conjugation with a bisphosphonate would possibly increase the chances of targeted drug delivery. Our current strategy was to conjugate zoledronic acid with multiple drugs loaded PLGA NPs to focus on the state-of-the-art development of active targeting of drug to bone tissue. From the bone binding assay, it was evident that the bone binding was time dependant and that bisphosphonates binding to the bone was more since osteoclasts were lacking in the human bone powder that may possibly interfere with the binding. The co-encapsulation was challenging since epirubicin is mildly hydrophobic in nature. But the lipophilicity of gemcitabine made up for their stability. The sizes of the PZ-1, PZ-2, PZ-3 NPs were in the range that may potentially avoid clearance from the bloodstream and the positive zeta potential values indicated towards a stable colloidal solution with uniform dispersion. Therefor what is attained is enhanced synergism. The micrographic images also hinted towards a uniform NP with no leakage or disrupted membrane. As seen from the sizes of PZ-3 under the influence of changing external pH, there were significant variations. T lower pH (3.5–5), the size of the PZ-3 did not show significant variations whereas at higher pH (6–8) the sizes drastically reduced. This may be attributed to the fact that at lower pH there is slow/sustained release of drugs whereas in higher pH an increased release of drugs. The rigidity of lipophilic structure of the NPs at lower pH makes it more stable, hence the controlled release of drugs. At higher pH the mobility of the drug molecules increased due to augmented hydrophilicity which causes drug release and decrease in size. Hence PZ-3 may be correctly assumed as pH-sensitive. This promises a very encouraging approach since the tumor microenvironment generally does not exceed pH 5. The high entrapment efficiency (91%) of PZ-3 was may be a result of the lipophilicity of gemcitabine which overcame the mild hydrophobicity of epirubicin and allowed slow diffusion of the drugs across the membrane of PLGA-ZOL NPs. This may be excellent for slow and sustained targeted release of drugs as may be seen from the in-vitro studies. It was clearly noticed from the in-vitro release readings that the measured release of drugs from NPs was over a longer time length in acidic pH than normal physiological pH. This may be better for future therapeutic applications since tumor pH in majority of cases rarely exceed 5. Furthermore this progressive release of drugs may also be attributed to their hydrophobicity and interaction within the NPs.

The achievement of nanoparticle drug delivery along with conjugation of a bisphosphonate would entirely depend upon the cytotoxicity of the drug delivery system within the cancerous cellular environment. Hence, cytotoxicity studies formed a crucial part of our study where we performed the MTT assay, live dead assay and Alamar blue assay. Alamar blue assay was chosen since it is a simple one-step assay, may be utilized for a large screening and the values in absence of fluorescence microscopy may be colorimetrically measured. All the studies revealed that the MG-63cell growth was inhibited with PZ-1, PZ-2 and PZ-3. However, the cell growth was least on treatment with PZ-3 owing to the synergistic effects of multiple drugs. It was noted that the drug concentration around the IC_50_ dosage resulted in maximum cell death. Too dilute samples hardly resulted in any cytotoxicity while too much concentration resulted in cell toxicity in the surrounding cellular environment which is not desirable. The treatment of primary bone cells with PZ-1, PZ-2 and PZ-3 exhibited cytocompatibility comparable to the PBS since the multiple drugs encapsulation within an organic nanoparticle may have resulted in a cytocompatible product than free drugs. Increased tumoricidal activity of the PZ-3 indicated towards better encapsulation and targetivity of the nanocarriers as compared to the standard formulations. This interesting find was consistent with that of the findings of the in-vitro studies. Reversion of the well-grown tumors may be due to the effect of the release of tumoricidal drugs which were co-loaded in the NP vehicle. There was a criterion where the best results were acquired with treatment of PZ-1, PZ-2 and PZ-3 within 10–14 days of development of tumor since they weren’t particularly large. The pathway for the anti-tumoral action of the PZ NPs remains unclear but diminished size of the tumors may be related to the possible instigation of macrophages and cytokines^40^.

Cellular uptake studies indicated increased uptake of NPs rather than the free drugs. This may be attributed to conjugated zoledronic acid which has affinities towards bone cells. This conjugation made for ideal nanocarrier for targeted delivery of drug to the bone tumor tissues. The increased uptake of drugs by MG-63 cells may escalate the therapeutic efficiency by causing increased cell apoptosis as a result of DNA damage. The progression of increased cellular uptake in case of NPs may be due to the small size which led to improved endocytosis. This benefits easy internalization of NPs within the cells thereby increasing cellular uptake efficiency. Nonetheless, to oversimplify the process of internalization of NPs by cells is foolhardy when the procedure is almost non-existent. But NPs having a size < 100 nm may be taken up by the cells via endocytosis. The surface charge on the NPs also has a significant role in the process. Rapid internalization of NPs with a positive charge was generally the accepted fact.

## Conclusion

Our study presents the synthesis of nanoparticle drug delivery systems which resulted in a more encouraging pharmacokinetic profile with increased circulation time that could possibly augment the drug uptake and accumulation via passive diffusion method. The PLGA NPs were chosen since they already possessed significant features such as tremendous cytocompatibility and biodegradability. The novel feature of the multiple drugs loaded NPs synthesized was the conjugation with zoledronic acid which had a high affinity towards the bone cells which caused enhanced cellular uptake of the NPs by MG-63 cells. Another important point was the co-encapsulation of multiple drugs within the NPs which resulted in increased tumor regression in mice. Therefore, we can safely conclude that a novel targeted biocompatible NP has been created which through the initial studies has been proven to be extremely successful. However the transition of the PZ-3 NPs from laboratory bench to clinical trials may be subjected to still more rigorous research which may be undertaken. The evaluation of a natural/organic substance as a major component of NPs has been previously done. Nonetheless, its conjugation with a bisphosphonate along with multiple drugs as a potential treatment for osteosarcoma may be furthered in future.

## Supplementary information

Supplementary Figure.
