# Supplementary material for: A multiple drug loaded, functionalized pH-sensitive nanocarrier as therapeutic and epigenetic modulator for osteosarcoma
Source: Sci Rep. 2020 Sep 23;10:15497. doi: 10.1038/s41598-020-72552-z (PMC7511925; doi:10.1038/s41598-020-72552-z)
Supplement: Supplementary file 1 — Supplementary Figure. [file 41598_2020_72552_MOESM1_ESM.pdf]

A multiple drug loaded, functionalized pH-sensitive nanocarrier as therapeutic and epigenetic modulator for osteosarcoma

Ye Yuan, Jia-Xing Song, Mei-Na Zhang, Bao-Shan Yuan*

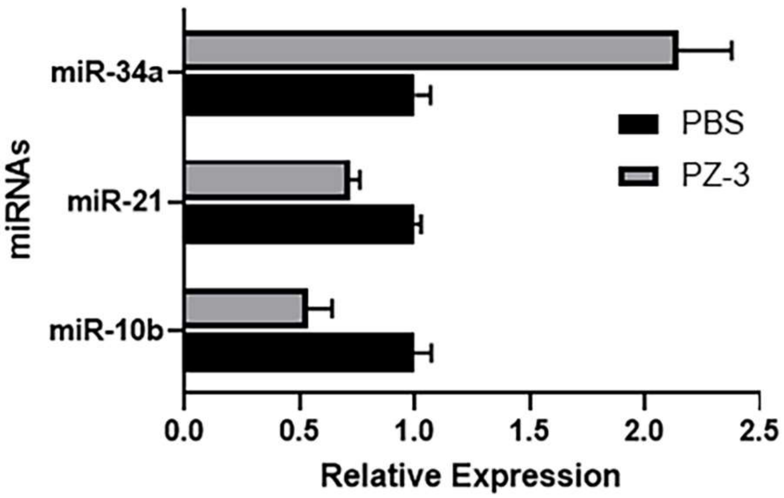

Supplementary Figure 1: miRNA levels detected in tumors obtained from rats treated with PBS and PZ-3.
